# Controversy in the Management of Cholangitis Secondary to Hydatid Daughter Cysts

**DOI:** 10.1155/1991/58262

**Published:** 1991

**Authors:** Brian Davidson, T. Ezaki, Nagy Habib

**Affiliations:** Hepatobiliary and Liver Transplantation Unit University Department of Surgery Royal Free Hospital and School of Medicine Pond Street London NW3 2QG UK

## Abstract

A 36 year old Cypriot woman, resident in the U.K. since the age of three years, presented with pyrexia, jaundice and upper abdominal pain. On ultrasound examination the biliary tree was dilated, contained sludge and a cystic lesion was present in the liver. An endoscopic cholangiogram showed multiple filling defects in the bile duct which were not felt to be removable endoscopically and a nasobiliary drain was therefore inserted. On resolution of the cholangitis with drainage and antibiotics a laparotomy was performed. The right lobe of the liver was largely replaced by a multiloculated cyst and the bile duct contained multiple hydatid daughter cysts. A right hepatectomy was performed with t-tube drainage of the evacuated bile duct. She made an uneventful recovery and has had no problems on subsequent follow up. Histology confirmed an intrabiliary rupture of a hydatid liver cyst.

Cholangitis secondary to daughter cysts is a rare but recognised complication of hydatid liver cysts. Management of hydatid liver cysts by formal resection is controversial but may be preferable in this situation.


*HPB Surgery*, 1991, Vol. 4, pp 321–329
Reprints available directly from the publisher
Photocopying permitted by license only

© 1991 Harwood Academic Publishers GmbH
Printed in the United Kingdom

### CASE REPORT

### CONTROVERSY IN THE MANAGEMENT OF CHOLANGITIS SECONDARY TO HYDATID DAUGHTER CYSTS

BRIAN DAVIDSON, T. EZAKI and NAGY HABIB
*Hepatobiliary and Liver Transplantation Unit, Royal Free Hospital and School of Medicine, Pond Street, London, UK*

*(Received 7 May 1991)*

A 36 year old Cypriot woman, resident in the U.K. since the age of three years, presented with pyrexia, jaundice and upper abdominal pain. On ultrasound examination the biliary tree was dilated, contained sludge and a cystic lesion was present in the liver. An endoscopic cholangiogram showed multiple filling defects in the bile duct which were not felt to be removable endoscopically and a nasobiliary drain was therefore inserted. On resolution of the cholangitis with drainage and antibiotics a laparotomy was performed. The right lobe of the liver was largely replaced by a multiloculated cyst and the bile duct contained multiple hydatid daughter cysts. A right hepatectomy was performed with t-tube drainage of the evacuated bile duct. She made an uneventful recovery and has had no problems on subsequent follow up. Histology confirmed an intrabiliary rupture of a hydatid liver cyst.

Cholangitis secondary to daughter cysts is a rare but recognised complication of hydatid liver cysts. Management of hydatid liver cysts by formal resection is controversial but may be preferable in this situation.

KEY WORDS: Hydatid disease, liver cyst, cholangitis, hepatectomy, endoscopic sphincterotomy

### INTRODUCTION

Hydatid disease is uncommon in the United Kingdom, most often being found as an asymptomatic cyst within the liver[1]. Cyst rupture may occur into the biliary tree producing cholangitis[2]. Rarely this may be due to occlusion of the common bile duct by daughter cysts[3,4]. We report on such a case and discuss the controversies involved in its management.

### CASE REPORT

A 36 year old Cypriot lady, resident in the UK since the age of three years, presented to the Out-Patient clinic with a five day history of colicky right upper quadrant pain which was exacerbated by eating and which radiated through to her back. There were no other symptoms on systemic enquiry. She was five weeks post partum with her fifth child and had been found to be hepatitis B surface antigen

Address correspondence to: Mr. Brian Davidson, University Department of Surgery, Royal Free Hospital, Pond Street, London NW3 2QG, UK

positive during her fourth pregnancy. There was no other significant past medical history. On clinical examination she was pyrexial, mildly icteric with tenderness and guarding in the right upper quadrant of the abdomen.

Ultrasonography showed the presence of dilated intra and extrahepatic ducts with sludge in the common bile duct. The gallbladder was dilated and contained a calculus. A multiloculated cyst was present in the liver compatible with hydatid disease. Abdominal CT scan confirmed a large cystic mass lying within the right lobe of the liver (Figure 1a) and a distended gallbladder containing a single calculus (figure 1b). Liver function tests on admission were bilirubin 20(5-17$\mu$mol/1), alkaline phosphatase 142(35-130) and aspartate transaminase 275(5-40). Urea and electrolytes, clotting screen and full blood count were within normal limits.

Endoscopic retrograde cholangen-pancreatography (ERCP) was carried out which confirmed the presence of large opacities within the common bile duct which were not felt to be amenable to endoscopic removal (Figure 2). A nasobiliary drain was left *in situ*.

Intravenous antibiotic therapy was commenced with a combination of mezlocillin (5g t.i.d.), metronidazole (500mg t.i.d.) and mebendazole (500mg t.i.d.).

At laparotomy a distended gallbladder was found containing a single calculus and the right lobe of the liver was largely replaced by a cystic mass. Operative cholangiography confirmed multiple opacities within the common bile duct (Figure 3). A cholecystectomy was performed, the common bile duct explored and multiple hydatid daughter cysts removed (Figures 4 and 5). The right lobe of the liver containing the cyst (Figure 6) was then resected in a right hepatectomy and a t-tube drain inserted in the common bile duct.

Pathology of the resected right lobe of liver confirmed a multiloculated hydatid cyst communicating with the biliary tree.

Her post operative progress was uneventful and her t-tube was removed after a satisfactory cholangiogram. Liver function tests had returned to normal within two months of surgery with bilirubin 5 (5-17), alkaline phosphatase 81 (35-130) and aspartate transaminase 28 (5-40). Albendazole therapy was continued for three months. An abdominal CT scan which was performed at one year following surgery showed no evidence of residual disease and she has remained asymptomatic for a follow up period of 18 months.

### DISCUSSION

Rupture of the liver hydatid cyst into the biliary tree presented in the early post partum in the present case. An association between cyst rupture and trauma has previously been suggested[4] and the trauma involved in delivery may therefore be implicated. In addition there is some evidence of accelerated liver hydatid cyst growth during pregnancy, possibly related to the depression of cell mediated immunity[5].

The optimum management of a patient with cholangitis who is suspected of having hydatid liver disease must involve clearance of the common bile duct, eradication of the primary liver cyst and the prevention of recurrence.

The least invasive management possible would involve endoscopic sphincterotomy (ES) for clearance of the common bile duct[6] and subsequent drug therapy for eradication of the liver cysts. Unfortunately the currently available drug therapy,

(a)

(b)

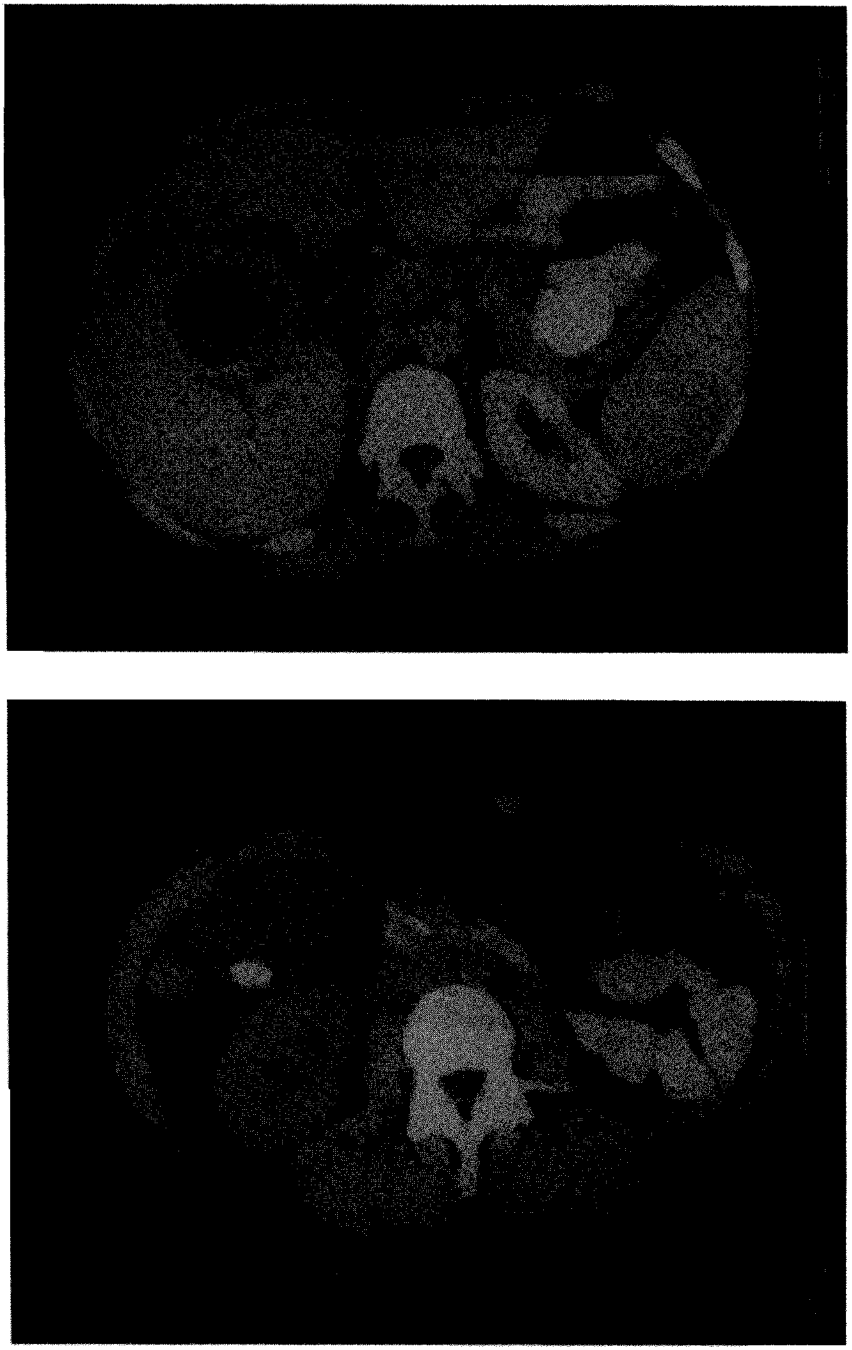

**Figure 1** Abdominal CT scan. The right lobe of the liver contains a multiloculated cystic mass (a) and a single gallstone is seen within a grossly dilated gallbladder (b).

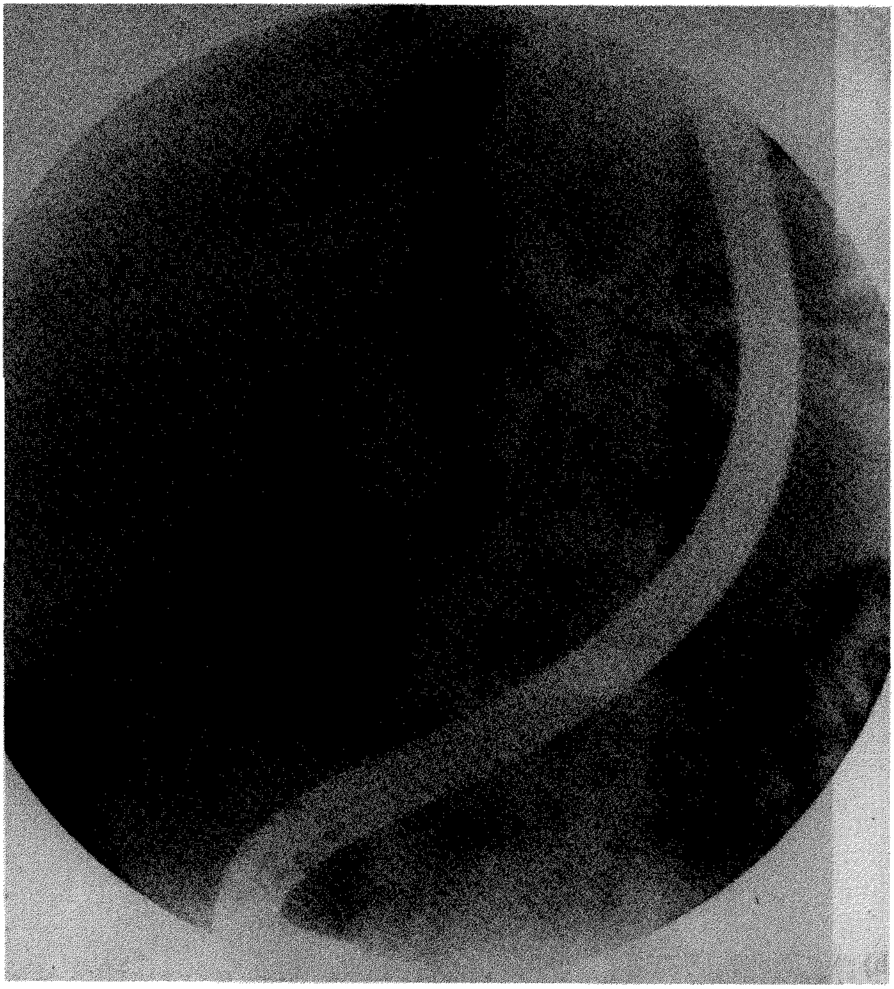

**Figure 2** Endoscopic retrograde cholangiography. The endoscopic cholangiogram showed multiple filling defects within the common bile duct.

although showing satisfactory penetration into hydatid cyst fluid and eradication of some hydatid daughter cysts[7], has not yet been established as an alternative to operative resection for established liver cysts. Surgical intervention is therefore required independent of pre-operative common bile duct clearance. Pre-operative ES may also have the disadvantage of introducing pathogens into the biliary tree promoting post-operative sepsis[2].

Although small asymptomatic liver hydatid cysts may be treated expectantly[8] those that are complicated by fistulae require surgery. Fistulae into the biliary tract may occasionally present with cholangitis without mechanical obstruction of the

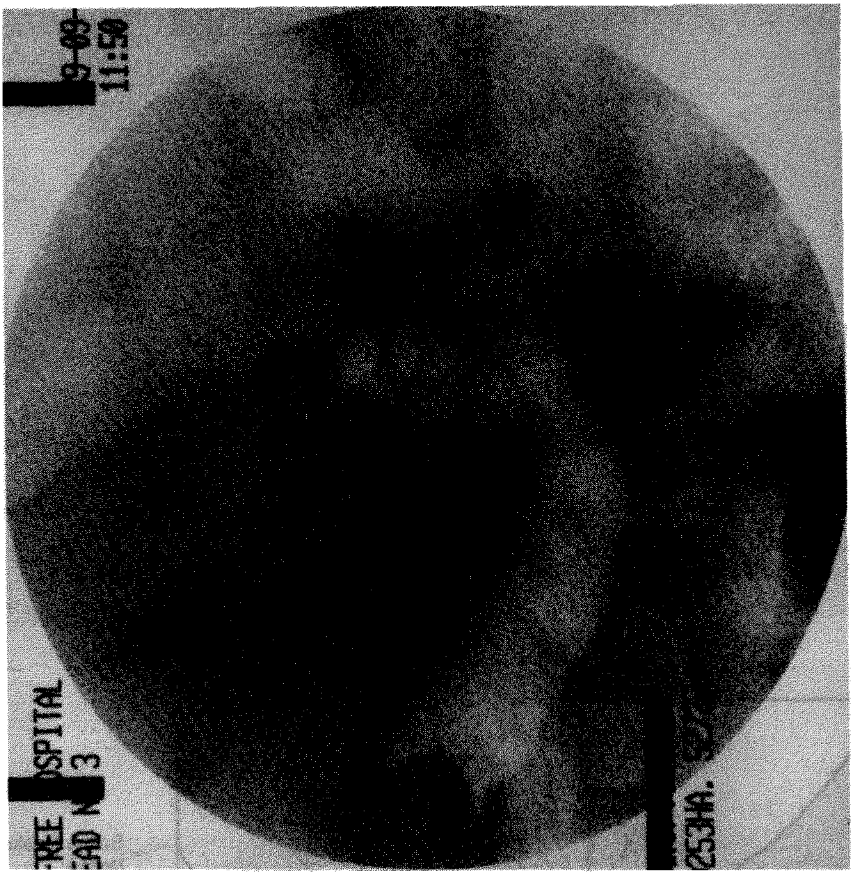

**Figure 3** Operative cholangiogram. Operative cholangiogram showing a dilated common bile duct with multiple filling defects.

biliary tree[2]. Operative cholangiography allows both the presence of daughter cysts in the common bile duct to be detected and the site of communication with the parent hydatid cyst to be established. If the common bile duct is explored for the removal of daughter cysts and the mother cyst is not removed a surgical drainage procedure such as a sphincterotomy or choledochoduodenostomy is generally carried out to prevent further bile duct obstruction[2,3]. Whether this may lead to hepatic reinfection with further cyst formation has not been established. In the present case removal of the parent cyst avoided the need to carry out permanent drainage of the biliary tree and a t-tube drain alone was left in the common bile duct to allow post operative cholangiography.

In addition to controversy over the management of cholagitis secondary to hydatid daughter cysts the type of surgery employed for the mother cyst is also controversial. The earlier reports on surgical management by means of tube drainage and marsupialisation of the cysts had unacceptable levels of chronic

(a)

(b)

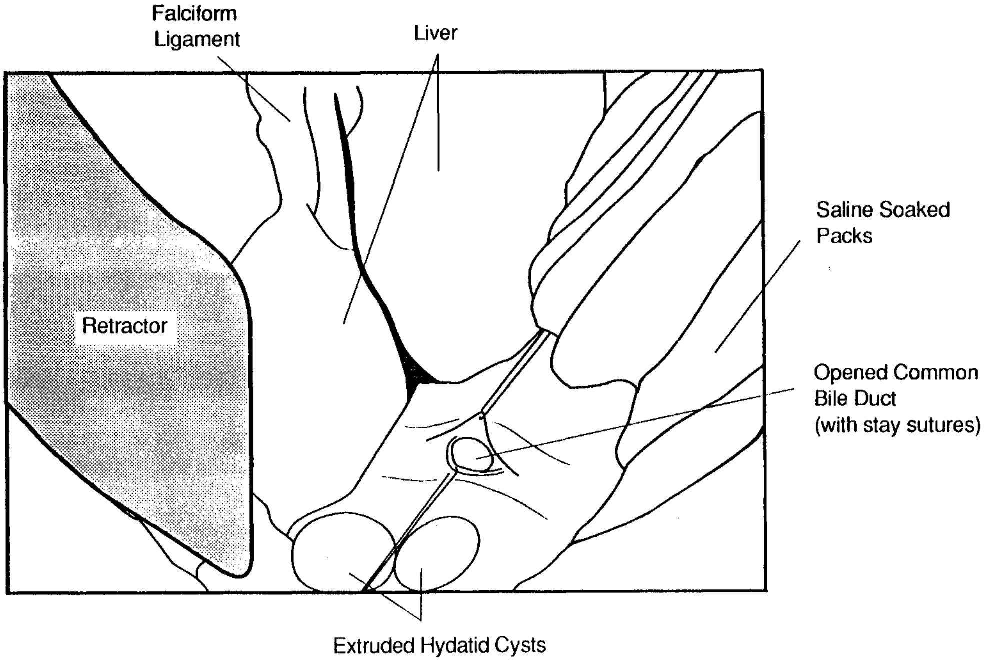

**Figure 4 (a and b)** Choledochotomy. The exploration of the common bile duct is demonstrated along with the extruded hydatid daughter cysts. The surrounding tissues are covered with saline soaked packs to prevent implantation of spilled scolices. (*See colour plate at the back of this issue*).

**Figure 5** Daughter cysts. The hydatid daughter cysts following removal from the common bile duct. (*See colour plate at the back of this issue*).

sepsis, haemorrhage and biliary fistulae and have been superceded by omentoplasty[9] or pericystectomy[10]. Omentoplasty appears to be a safe procedure which is applicable to the majority of hepatic hydatid cysts whereas pericystectomy may be complicated by major haemorrhage and is inadvisable if the cyst cavity is in the vicinity of the hepatic veins or the inferior vena cave[11].

Hepatic resection is felt by some to be too radical a procedure to carry out for benign disease[3]. Others, however, have shown it to be a safe and effective management in selected cases[10]. It seems likely that hepatic resection, as carried out in the present case, is justified only in centres specialising in liver surgery where an acceptable level of morbidity and mortality would be expected from this procedure.

Despite the many publications in the field of hydatid liver disease the optimal management remains unclear and requires further carefully analysed studies.

### *References*

1. Stallbaumer, M.F., Clarkson, J.M., Pritchard, J.F., Bailey, J.W. and Morris, D.L. (1983) Serological diagnosis and current epidemiology of hydatid disease in England and Wales. *Gut*, **24**, A996
2. Dawson, J.L., Stamatakis, J.D., Stringer, M.D. and Williams, R. (1988) Surgical treatment of hepatic hydatid disease. *Br.J.Surg.*, **75**, 946–950
3. Barros, J.L. (1978) Hydatid disease of the liver. *Am.J.Surg.*, **135**, 597–600
4. Al-Hashimi, H.M. (1971) Intrabiliary rupture of hydatid cyst of the liver. *Br.J.Surg.*, **58**, 228–232

(a)

(b)

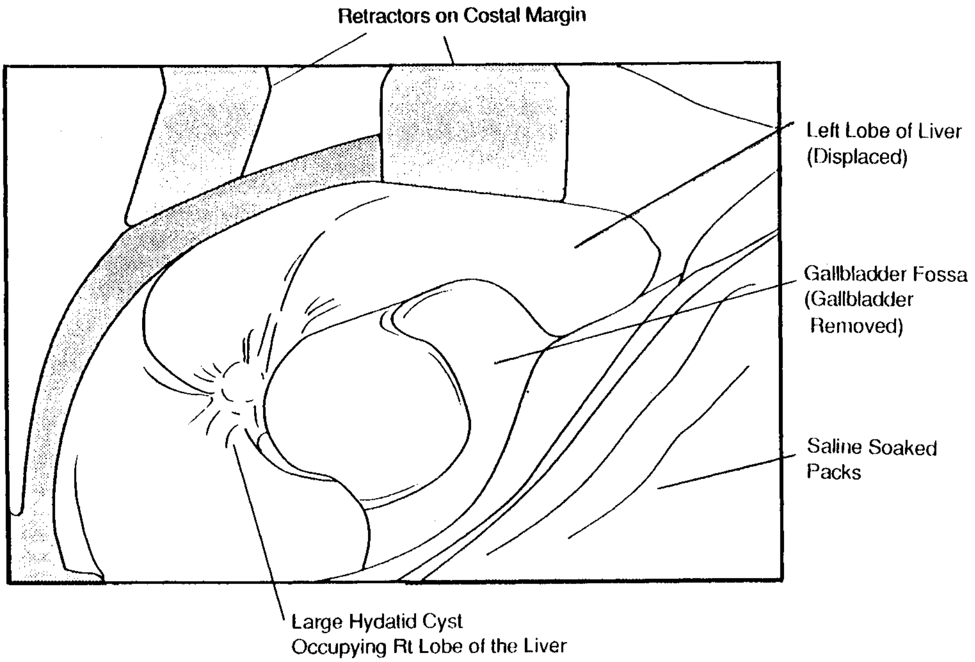

**Figure 6 (a and b)** The hepatic cyst. The majority of the right lobe of the liver was occupied by a large cystic mass. (*See colour plate at the back of this issue*).

5. Kain, K.C. and Keystone, J.S. (1988) Recurrent hydatid disease during pregnancy. *Am.J. Obstet. Gynecol.*, **159**, 1216–7
6. Vignote, M.L., Mino, G., de la Mata, M., de Dios, J.F. and Gomez, F. (1990) Endoscopic sphincterotomy in hepatic disease open to the biliary tree. *Br.J.Surg.*, **77**, 30–31
7. Morris, D.L. (1987) Pre-operative albendezole therapy for hydatid cyst. *Br.J.Surg.*, **74**, 805–806
8. Saidi, F. (1982) In: Calne, R.Y. (Ed) *Liver Surgery*, pp. 61–74. Padua: Piccin Medical Books
9. Papadimitriou, J. and Mandrekas, A. (1970) The surgical treatment of hydatid disease of the liver. *Br.J.Surg.*, **57**, 431–433
10. Belli, L., del Favero, E., Marni, A. and Romani, F. (1983) Resection versus pericystectomy in the treatment of hydatidosis of the liver. *Am.J.Surg.*, **145**, 239–242
11. Little, J.M. and Deane, S.A. (1988) Hydatid disease. In: Blumgart, L.H. (Ed), *Surgery of the liver and biliary tract,* Vol. 2, pp. 955–966. London: Churchill Livingstone

*(Accepted by S. Bengmark 15 May 1991)*

